# Are We Asking Too Much of the Health Sector? Exploring the Readiness of Brazilian Primary Healthcare to Respond to Domestic Violence Against Women

**DOI:** 10.34172/ijhpm.2020.237

**Published:** 2020-12-08

**Authors:** Ana Flávia Pires Lucas d’Oliveira, Stephanie Pereira, Loraine J. Bacchus, Gene Feder, Lilia Blima Schraiber, Janaina Marques de Aguiar, Renata Granusso Bonin, Cecilia Guida Vieira Graglia, Manuela Colombini

**Affiliations:** ^1^Departamento de Medicina Preventiva, Faculdade de Medicina, Universidade de São Paulo, São Paulo, Brazil.; ^2^London School of Hygiene & Tropical Medicine, London, UK.; ^3^Centre for Academic Primary Care, Bristol Medical School, University of Bristol, Bristol, UK.

**Keywords:** Domestic Violence, Gender Based Violence, Primary Healthcare, Health System Readiness, Policy-Makers, Brazil

## Abstract

**Background:** There is growing recognition of the health sector’s potential role in addressing domestic violence (DV) against women. Although Brazil has a comprehensive policy framework on violence against women (VAW), implementation has been slow and incomplete in primary healthcare (PHC), and little is known about the implementation challenges. This paper aims to assess the readiness of two PHC clinics in urban Brazil to integrate an intervention to strengthen their DV response.

**Methods:** We conducted 20 semi-structured interviews with health managers and health providers; a document analysis of VAW and DV policies from São Paulo and Brazil; and 2 structured facility observations. Data were analysed using thematic analysis.

**Results:** Findings from our readiness assessment revealed gaps in both current policy and practice needing to be addressed, particularly with regards to governance and leadership, health service organisation and health workforce. DV received less political recognition, being perceived as a lower priority compared to other health issues. Lack of clear guidance from the central and municipal levels emerged as a crucial factor that weakened DV policy implementation both by providers and managers. Furthermore, responses to DV lost visibility, as they were diluted within generic violence responses. The organizational structure of the PHC system in São Paulo, which prioritised the number of consultations and household visits as the main performance indicators, was an additional difficulty in legitimising healthcare providers’ time to address DV. Individual-level challenges reported by providers included lack of time and knowledge of how to respond, as well as fears of dealing with DV.

**Conclusion:** Assessing readiness is critical because it helps to evaluate what services and infrastructure are already in place, also identifying obstacles that may hinder adaptation and integration of an intervention to strengthen the response to DV before implementation.

## Background

**Key Messages**

** Implications for policy makers**

- Assessing health system readiness to domestic violence (DV) is critical to understand and reduce preparedness gaps and anticipate potential challenges to ensure effective implementation of a new intervention.
- While having a policy and a regulatory framework on DV is crucial, political support and policy consistency and clarity are essential for implementing DV response in primary healthcare (PHC).
- Having adequate DV training and a supportive management are prerequisites for implementing DV care.
- Having a performance indicator on DV will ensure prioritization and visibility of DV care but it depends on the implementation and monitoring of key conditions for ensuring quality DV care.

** Implications for the public**

Findings from this study highlight how primary care clinics may be crucial to identifying cases, providing non-judgmental and confidential support, orientation and referral to women. Furthermore, service and systems level challenges affect the quality of domestic violence (DV) care offered by providers. Providers need managers’ and institutional support to have protected time with DV patients, safety protocols, adequate training, and continuous supervision. By strengthening health systems to respond to DV, service users will be able to receive quality care which may increase their trust in services and healthcare providers’ respect for confidentiality. Over time, this can help to decrease the adverse health consequences and enhance the comprehensiveness of primary healthcare (PHC) response to DV.

Violence against women (VAW) is a challenge to global public health and clinical services. Worldwide, 35% of women have experienced either physical or sexual intimate partner violence or non-partner sexual violence.^1^ VAW is associated with adverse physical and mental health outcomes for women^2^ who consequently use health services more frequently.^3^

 There is growing recognition of the health sector’s potential role in addressing VAW^4,5^ as the first entry point for women seeking help in many high, as well as in low- and middle-income countries (LMICs).^6^ Furthermore, despite new evidence on promising health interventions to address VAW in LMICs,^7^ system level challenges affect their implementation and effectiveness in primary healthcare (PHC).^8^ There is a growing recognition that implementation of these interventions requires attention to the readiness of healthcare systems to integrate a response to VAW into routine care, identifying gaps in structure, policy, and practice. Analysis of healthcare system readiness may also help understand why specific interventions may be effective in one context but not in others.^9^

 Readiness is not a new concept, but has been used in various healthcare contexts to refer to: (*a*) individual providers’ ability to respond to a specific health issue^10^; (*b*) service readiness to assess availability, performance and quality of VAW care offered^11^; (*c*) organizational readiness to implement a particular innovation.^12^ However, none of these tools offer a framework to assess systems’ capacity and preparedness for adopting a new intervention. For the purpose of this study, we used an adapted systems readiness framework focusing on processes that are needed to ensure system change for the implementation of a new intervention.^9^

 Brazil is characterised by a culture of violence and inequality that is linked to historical, political, economic, and social conditions.^13,14^ It has the fifth highest rate of female homicide in the world^15^ and one in three women have experienced sexual or physical violence by a partner.^16-19^

 In accordance with international agreements aiming to end VAW^20-24^ of which Brazil is a signatory, and also as a result of an active feminist movement, the government has developed a comprehensive legal and regulatory framework on VAW. The “Maria da Penha Law”^25^ is a key legal landmark. Following the law, the National Policy for Tackling VAW, developed by the Secretariat of Policies for Women in 2007 and 2011, established funding agreements between the different levels of public administration (Federal, States, Municipalities) linked to VAW policy implementation. This policy sharply increased the number of VAW specialized services all over the country.^26,27^ Despite this legal framework, the political prioritisation of VAW has diminished since 2016 along with the parliamentary coup in the country that ousted the elected president. In the same period, the Secretariat of Policies for Women, the main governing body on VAW, was abolished at Federal and at many municipal levels, including São Paulo city, and the national and local budget dedicated to VAW was drastically reduced.^28^

 Brazil has a National Health System (SUS) since 1988, offering universal and free care for all. The system prioritized PHC and adopted the Family Health Strategy policy to structure the health system.^29^ VAW entered the health policy agenda in Brazil mainly through an increased focus on sexual violence and the need for legal abortion in public hospitals in cases of rape or life-threatening risk.^30,31^ In this study we focused on the PHC response to the most prevalent form of VAW, domestic violence (DV), defined by the Maria da Penha law as “gender based violence perpetrated by a family member, someone who lives in the same house or any person with an intimate affective relationship independent of cohabitation.”^25^

 DV remains invisible within PHC,^32,33^ despite its many negative health outcomes^3,34,35^ and notwithstanding new health policies on VAW, such as compulsory reporting of all cases of VAW to epidemiological surveillance^36^ as well as the inclusion of the healthcare response to domestic and sexual violence in the Women’s National Health plan.^30^ The implementation of these policies, however, is slow and incomplete in PHC.^37^

 This study uses Brazil as a case study to assess the readiness of 2 PHC units to integrate an intervention to strengthen the response to DV and explore relevant systems challenges and facilitators.

## Methods

 Semi-structured interviews combined with facility observations and document analysis, constitute the formative phase of Healthcare Responding to Violence and Abuse, a programme of research that aimed to develop, implement and evaluate an intervention geared towards improving the care provided by PHC in Brazil to women exposed to DV.

###  Study Setting

 The research was conducted in two PHC Clinics in São Paulo city, chosen by regional managers because they were representative of the clinics in the region and had a violence prevention nucleus (NPV), which was responsible for coordinating care in cases of DV within the PHC Clinic. Situated in the southeast of Brazil, São Paulo is the most populous (12 million people) and richest city in the country. It is a city of contrasts, having many *favelas* (slums) and wealthy districts. About 50% of the population depend exclusively on SUS,^38^ while among women aged 15 to 45 years old using PHC clinics, around 45% reported experiencing sexual or physical violence from a partner.^39,40^

 As part of the Municipal Health Department, the municipal PHC clinics are free at point of use and managed by a range of private and non-profit organizations in each region of the city. The differences between the clinics in terms of service administration (public and/or private and non-profit organizations) were not analysed in this paper. See Table 1 for the characteristics of the two facilities we studied.

**Table 1:** Key Baseline Characteristics of the Study Clinics

	**PHC Clinic 1**	**PHC Clinic 2**
Location and covered area	Downtown area Cover 58 541 people in 27 159 households: ♦ 573 (2.1%) in collective tenements ♦ 42 (0.15%) lacking basic sanitation	Peripheral region Covers 43, 429 people in 13 348 households: ♦ 4851 (36.3%) in favela areas ♦ 1778 (13.3%) lacking basic sanitation
Service administration	Fully administered by a private and non-profit organization Three teams of family medicine Area covered by gynaecologists and obstetricians, paediatrician and general medicine Street team for homeless access to the clinic	Mixed administration: half by direct municipal administration (public employees) Three teams of family medicine managed by a private and non-profit organization Area covered by gynaecologists and obstetricians, paediatrician and general medicine
Staff	118 employees: 94 women, 24 men	79 professionals: 63 women, 16 men
DV cases reported to the mandatory surveillance in 2017	3	3
NPV members	1 Social worker, 1 psychologist, 1 nursing technician	1 Social worker
Any regular meetings at the facility^a^	Yes	No

Abbreviations: PHC, primary healthcare; NPV, violence prevention nucleus; DV, domestic violence. ^a^ Any sort of regular team meeting among the providers to discuss any aspect of the work in the PHC clinics.

###  Study Methods and Data Collection

 To assess the health systems readiness within our study settings, we used an adapted readiness framework,^41,42^ which was pilot-tested in Palestine.^9^ We consider health systems as open, complex and adaptive systems influenced by both policy-makers and people working and interacting with them (eg, patients, health providers and communities).^43^ The adapted readiness framework explored the following health systems dimensions (based on mainstream health systems frameworks, including the World Health Organization (WHO) building blocks)^6^: governance and leadership; resources and infrastructure; information and documentation; values and beliefs; service delivery; health workforce and coordination.

 To explore the health systems readiness of our study settings, we conducted: (*a*) document analysis of VAW policies from Health Departments and Women’s Secretariats from São Paulo and Brazil; (*b*) 2 structured facility observations of the study clinics to collect information on service delivery and workforce, institutional framework, infrastructure and medical supplies; and (c) 20 semi-structured interviews with the 4 health managers and 16 health providers – 10 in PHC Clinic 1 and 6 in PHC Clinic 2 (Table 2).

**Table 2:** Background Characteristics of Interviewed Managers and Providers (n = 20)

**Variable**	
Gender	
Male	4
Female	16
Age	
Range	24-70
Median	42
Profession	
Doctor	6
Manager	4
Nurse	3
Community health agent	3
Social worker	2
Psychologist	1
Nursing technician	1
NPV member	
Yes	4
No	16

Abbreviation: NPV, violence prevention nucleus.

 The document analysis of policy and regulatory records around VAW included 12 health policy laws and guidelines, and 17 VAW and DV municipal or federal laws. Documents were retrieved from governmental online platforms. We did not limit the review to DV cases, including also VAW to generate a broader understanding of the context as different types of violence were often interlinked. Relevant information was extracted from the policy documents and organised in a matrix with details of year, authorship, type, definition of violence, use of gender concept and proposed PHC response. Information produced by this review informed the analysis in relation to the governance and leadership systems’ dimensions.

 A structured facility-observation was conducted at each study clinic using a adapted facility checklist tool that was field-tested by local researchers prior to its implementation.^41,42^ The checklist tool was divided in three sections: (1) Service delivery and health workforce; (2) Institutional framework (governance at facility-level) and financing; and (3) Infrastructure, medical equipment, and supplies. Two researchers deductively coded data using the dimensions from the readiness framework. Information from the facility observations were subsequently summarised into tables and informed the analysis regarding infrastructure and service delivery themes.

 The semi-structured interviews were conducted between October and December 2017. Interview participants were selected purposively based on their professional occupation and level of interest and involvement with violence response according to the local manager, aiming for diversity. The interviews were conducted in Portuguese by 4 female co-authors (SP, JMA, RGB, CGVG), who are also health professionals (nurses and psychologists), but outside the clinics under study.

 Interviews took place in a private location inside the study clinics and lasted on average 1 hour (ranging from 40 minutes to 3 hours). Interviews explored knowledge of DV policies and procedures, experiences with DV identification and referral, knowledge of specialized DV services, and values and beliefs about DV. In addition, managers were asked about their views and experiences with policy implementation of DV.

 Upon written consent, the interviews were recorded, and subsequently transcribed verbatim into Portuguese. Eight interviews were also translated into English to facilitate data analysis workshops with UK collaborators (MC, LJB). Thematic analysis^44,45^ was undertaken using NVivo 11 to manage the data. The interviews were read and annotated separately and afterwards discussed within the team to identify recurring patterns in the data and develop an initial coding frame. Coding was both deductive, drawing on dimensions of the readiness framework, but also inductive allowing new ideas to emerge from the data.^44,45^ Subsequent interviews were double coded in Portuguese by the Brazilian research team and the codebook was refined as further interviews were analysed. In order to facilitate analysis, those reports synthesizing the best quotes for each code, were organised by professional groups to help explore data across codes and within the dimensions of the health system readiness framework. We did not analyse the data by professionals’ categories in this paper.

 Data from the facility observations and the policy documents were first analysed separately and then integrated into the analysis of the qualitative interviews in order to triangulate findings, especially in relation to governance and leadership, service delivery and infrastructure. The research team held meetings to discuss interpretation of the summarized data of interviews, documents, and facility observation to identify key themes that cut across building blocks and sub-themes, generating the three crossing the mes as an interpretative synthesis.

 Our analysis of the health systems readiness focused on both policy content and policy implementation.^7,46^ We organised our findings around three main overarching crosscutting themes: Health policies on DV; Health services organization and Health workforce.

## Results

###  Health Policies on VAW: Loose Governance and Limited Leadership

**Figure 1:**
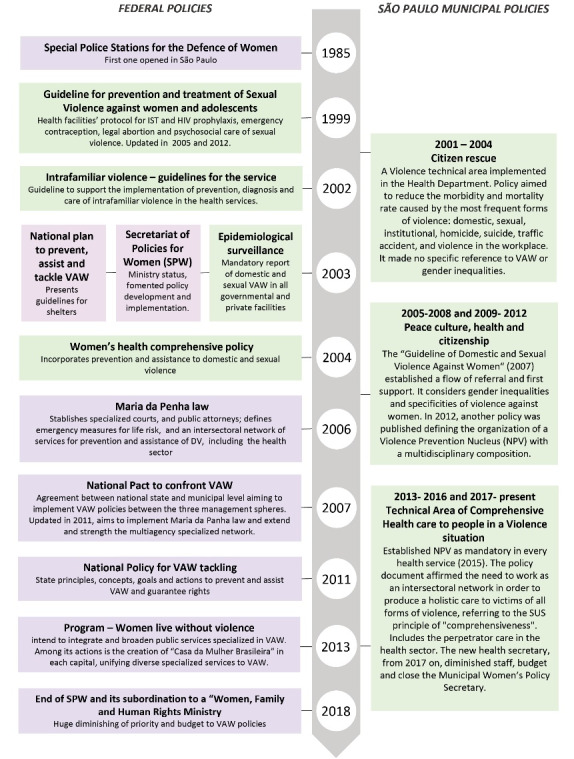
Brazil’s and São Paulo City Policies Regarding Domestic VAW. Note: The green boxes represent health policies that address VAW, while the purple boxes represent specific VAW policies. Abbreviations: VAW, Violence against women; NPV, violence prevention nucleus.

 The first crosscutting theme focuses primarily on two specific system dimensions: leadership and governance and financing issues. Our document analysis illustrates a comprehensive legal and policy response to DV in Brazil, particularly in São Paulo (Figure 1).

**Figure 2:**
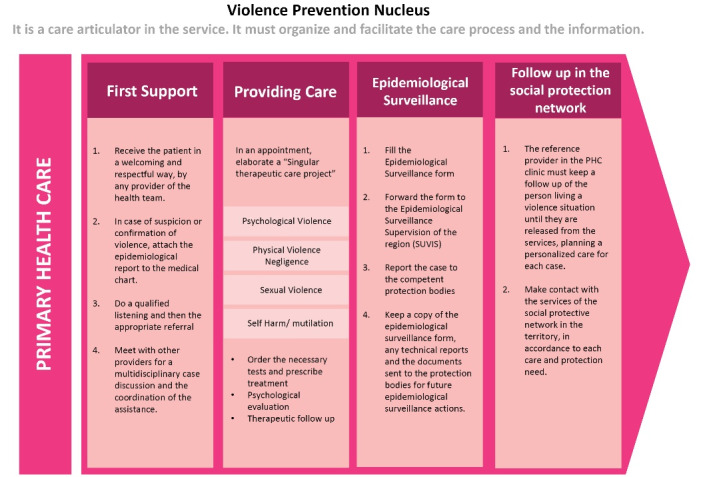
Flow Chart of the PHC for People Experiencing Violence (in Accordance With the Care Pathway Policy). São Paulo Municipality, 2015.^51^ Abbreviation:PHC, primary healthcare.

 Prior to the Maria da Penha’s law, at municipal level, the São Paulo Health Department formulated various policies on interpersonal violence since 2001.^47-51^ It created a Violence Technical Area (Figure 1) with a representative in each one of the 6 regions and 27 sub-regions of the city to implement municipal and federal guidelines on violence.^52,53^ In the 148-page municipal guideline^51^ on violence and health, VAW was briefly discussed in a special section on women’s health, although the emphasis was on sexual violence. DV was dealt within “interpersonal violence,” comprising a care pathway to children, elders, women, and men. The official care pathway for the PHC is illustrated in Figure 2.

 According to the municipal guidelines,^51^ each PHC facility should have a NPV responsible for DV training, epidemiological surveillance, support for DV cases and multiagency networking for all types of violence. All NPV members and representatives of the technical area for violence received mandatory monthly training from March 2016 to March 2018. Although NPV staff chose to work in an area dealing with violence, they have no designated time to engage in these activities, or additional salary, as observed through the interviews and facility observations.

 Our document analysis and interviews with health managers highlighted how the evolution of these policies was fragmented. The proposed policies changed each time a new mayor was elected, generating a new cycle of training and regulations without due consideration of the previous strategies, or monitoring progress.

*“Looking at the history of all this (policies on violence in the Municipal Health Department) I notice that the new discussion did not use the previous guidelines (...). So the guidelines on violence against women, children and adolescents and elders (from 2010-2011), with goals and care pathways, for instance were not used by the next guideline, which is the Pathway of care*” (Manager 1).

 The interviews with health managers highlighted the ambiguity in the DV policies, characterised by a lack of clarity and specificity at both municipal and/or regional levels, and generic roles, responsibilities and pathways of care laid out for healthcare providers and the NPV team. Except for one regional manager, the health managers had little knowledge and contact with DV specialized legal and psychosocial services, hampering multi-agency coordination.

 Furthermore, the PHC facility managers did not consider DV a priority, despite recognizing its impact on public health. Competing priorities such as common health problems, and other types of violence, such as violence against children or suicide, deserved more attention.

*“There are so many priorities here, that it is hard to say that violence is a priority. There is a priority of attention to dentistry, gynaecology, prenatal (care), so it is that old story, what do you need first? Rice and beans* ^[1]^ *, and then you’ll think about meat, (after you have the essential) Right? So, it’s a priority?, I do not know if it’s the top priority. (...) Look, between a woman’s violence and a suicide risk, we invest more in the suicide risk. Actually, the two have the risk of dying, right? But the risk of suicide is more, more shocking to us, you see? We have a lot of this here*” (Manager 3).

 In terms of financing, there is no budget dedicated to VAW or DV within the general budget for policy implementation of PHC clinics from the Municipal Health Department. Furthermore, the low prioritisation of DV reflected a broader organisational issue within the PHC structure, where contracts between the private non-profit organizations and the government are increasingly based on performance indicators (mainly the number of consultations) linked to payment – lacking performance indicators for any form of violence.

*“We are turning PHC into a dashboard of indicators. (…) When you have indicators, you mobilize the team and you make it happen; when you do not have indicators for something, it falls into limbo. (...) Because you have to deal with so many other things, that you miss some, you do not have time. (...) I think that before, when we did not have this contract … And, well, look, quantitative issues, they always existed. We always had attendance goals. This always existed. But you could have a margin to manoeuvre. So, if you allowed a professional to take a course, you could justify the week he did not attend because he was in training. We can’t justify this today*”(Manager 2).

###  Health Services Organization: Weakly Implemented Policies and Unsupportive Structure

 Another crosscutting theme concerned the weak implementation and dissemination of two specific elements of the municipal guidance at facility level: NPV and epidemiological surveillance. This theme articulates systems’ dimensions related to service delivery, information system, and touches upon health infrastructure problems.

####  Poor Implementation of the NPV Structure

 We found that the NPV policy was not fully implemented in the study clinics, despite NPV being described as “in place” in both (Table 1). NPV teams often felt they had no clear role or defined pathway of care, as there was no properly defined organizational structure to work with. In particular, some interviewers talked of heterogeneous flows and lack of knowledge of NPV teams among providers.

 Some NPV staff also reported feeling isolated and lacking support from other providers within their clinic and, more broadly, they felt that, no investment and visibility was given to DV from the municipal manager.

*“I find difficulties, yes, but I think that as a service, because it is something that you have to join the team, this issue of violence, I think that it should be invested like a campaign, like a more grandiose thing, that would bring visibility, because, it is an ant’s job, one to one, two or three people, in isolated places, telling, it is difficult to tell even more when the other doesn’t want to hear, to listen, it is very difficult*” (Nursing technician 1).

 Facility health managers were not proactively supporting the development of NPV teams and attributed its partial implementation to various factors including limited specialists, disinterested staff, staff turnover, and low awareness of the existence and role of the NPV within the clinics.

*“Look, at this moment it is difficult, okay? So, you can see that I’m physically rearranging the unit, but I believe that if I had an interested group, I could support them, right? I can support them. I do not know how far I would go” *(Manager 3).

*“I do not believe it [NPV] work. We have professionals who are sensitized, who are reference for that, but here we total 130 employees. I do not tell you that these 130 people know. They do not know (That NPV is in place)”* (Manager 2).

 The poor implementation of NPV policy also resulted in uncertainty about care and referral pathways in the clinics. Many providers mentioned the lack of a DV protocol with a clear flowchart explaining referral of DV cases.

*“We do not have a protocol, a northern star to guide us, we go more because, look … A flow chart. A flowchart. “Oh … in this case where will I refer her? When? Where do I take her? What do I do? I’m going back to where? Will you give it back? “Something like that, I think it would be interesting” *(Nurse 1).

####  Epidemiological Surveillance on Domestic Violence 

 Although most respondents knew about epidemiological surveillance in cases of VAW, its mandatory aspect and its purpose were misunderstood. Some of them confused it with a mandatory report to the police, while others perceived it to be an internal report needing patient consent.

*“Here we report and they forward it to the regional municipality and there they send it along, but how they do this?, I do not know. If they look for people … they should do a home visit, something like that”* (Nurse 2).

 This lack of clarity regarding epidemiological surveillance deterred some providers from reporting DV cases, as they feared repercussions by DV survivors’ family members.

*“It is mandatory to report, isn’t it? But what about the consequences? I mean, how are you going to protect yourself from them (family members)?” *(Community Health Agent 1).

 Providers’ fear for their own safety and the lack of clarity about recording DV information may account for the very low number of reported DV cases to epidemiological surveillance in 2017 (Table 1), especially when compared with the greater awareness of disclosed cases discussed in the interviews.

###  Health Workforce’s Values, Knowledge and Experiences Addressing Domestic Violence

 This last crosscutting theme articulates providers’ values and beliefs, health workforce and coordination. Five sub-themes are presented.

####  Views on Intimate Partner Violence as a Primary Healthcare Issue 

 Most providers recognized DV as a health issue, linked to physical symptoms such as chronic pain and vague complaints. DV impact on mental health also emerged strongly in the providers’ narratives, particularly symptoms and diagnosis related to depression and anxiety.

*“I think health professionals, especially those who are in primary care, especially family health strategy, I think they identify this as a health problem. […] I think they understand that this is one of our assignments”* (Manager 2).

*“I believe that here, with the experience that we have, the emotional component is the most important part. That is why I believe that violence also affects health, because it directly involves the person’s emotional state. It is what literally incapacitates*” (Community Health Agent 1).

 However, some providers still referred to DV as a disease needing to be “fixed by some kind of remedy” (diagnosis and treatment), regardless of the woman’s needs and choices. Women were often blamed for the violence due to lack of compliance with the healthcare providers’ recommendations.

*“If you think about it, violence is like a disease. If you are going to think comparatively, you want to heal that person from that. Will you be able to? Not always, sometimes the remedy you have to give is not enough if the person does not take it”* (Nurse 3).

 Many providers perceived their role as one of emotional support, depending on the women’s willingness to be helped. Some attributed a special role to PHC settings, described by some as the “kindest gate” being the first and easiest point of access to receive care. Some narratives also illustrated that healthcare providers had unrealistic expectations of “fixing” DV by changing the woman.

*“We are the entry point, theoretically. We are the people who have to listen to this violence; we are the people who have to notice it. (…) I think that health services are super powerful places to think about violence. They are places where they need to come, right?”* (Psychologist 1).

 “(…) *one of these days, I’m going to develop a tool which allows me to, enter this woman’s world and make her understand, that it is not normal. (…). I still have not been able to turn on this little switch and discover this tool to make her change, change her mind, to change her way of thinking” *(Nurse 1).

 The lack of municipal performance indicators for DV and the perceived high numbers of patients to be seen in order to meet the targets was an obstacle to DV identification and response among doctors and nurses, as they became anxious about time constraints.

*“(...) here we have fifteen minutes to attend each patient; we have the medical records, physical examination and everything else. To explore this *[DV]* is practically impossible, so it has to be very fast” *(Doctor 2).

####  Lack of Training Leading to Poor Staff Knowledge and Self-efficacy

 The majority of providers had not participated in any in-service training about DV, although NPV teams were supposed to offer training to all staff.

*“We have no training for this [DV]. We do not know how to approach the patient, how to act, what is most important to ask, where to refer patients, how we should give orientation... Because we were not trained. All the clinical part, we already know. ‘Patient has this, refer to that, ask this, do that.’ But for this aspect of aggression [DV], we have no preparation at all, none at all. (…)*” (Doctor 2).

 The lack of DV training also emerged as a reason underpinning the limited knowledge and awareness about how to deal with DV cases identified.

*“There is a huge lack of preparation, so, there is no way, (...) because the fact that you have a diploma doesn’t mean that you are prepared for all and any situation”* (Nursing technician 1).

 Despite the lack of training and clear guidance, some providers reported using a range of strategies and approaches when dealing with DV cases. Some actions were based on their professional and personal values and beliefs, while others were acquired through individual and colleagues’ experiences.

*“The patient sometimes comes with a complaint. She had never had high blood pressure and now she does. She has never had a stomachache and now she is dying of pain. I ask: ‘What’s going on at home?’ And they look at me, start to cry and sometimes do not talk. ‘Nothing is happening.’ ‘Then why are you crying? If nothing is happening, you do not have to cry.’ Some are able to go ahead and talk”* (Doctor 5).

 Many providers viewed DV as a complex issue rooted in strong traditional social and gender norms, which left them feeling impotent when facing DV cases.

*“So, while I feel good that I can help in some way, right? As a health professional, right? That makes me feel good, because somehow, I’m helping, right? Somehow. But I feel powerless. I feel totally impotent, because it is an impotence so huge..., you still live in a macho country, where the man still rapes his wife, beats her, thinks he has the right to do so, thinks that a woman is his property, that a woman is a punching bag, that he has the right to beat her, even though as a matter of fact he does not have this right. And we know that we have such weak laws in this country that little is resolved. So, you feel this way, very powerless”* (Nurse 1).

####  Fear for Own and Women’s Safety

 Another obstacle reported by providers when discussing their experiences of addressing VAW was fear for their own and women’s safety. Such fear was often reported as a reason for providers’ inaction towards identification and reporting cases, as mentioned earlier.

*“You have to pretend that you do not see. We end up pretending to be deaf, dumb and blind. It’s for avoiding everything to get worse. Not for us or for the women”* (Nurse 1).

 Some particularly feared family retaliation, as they remembered or heard about cases, which had put a provider’s life at risk.

*“That you have to go after it and treat that case. (...), the problem is that you become involved in some things that put you at risk. Here, I know that when I arrived, there was a doctor who had to ask for a transfer because he had been threatened with death. Would you stay in such a situation? You will not*” (Nurse 3).

####  Coordination

 Many providers were not aware of external referral services and how they worked, and most of them only knew about women’s police station and a counselling centre. Though many said they would like to have additional information about these and how to refer to them.

*“I do not know what the name is or what resources they work with, nothing at all. But I would like to know more. (...) I think it is essential that such work has articulation with outside reference centers, police in some cases, right?” *(Doctor 3).

 However, personal contact and direct communication between social workers and individuals from external agencies fostered respect for confidentiality and collaboration across sectors.

*“We contacted the DV center I went to a meeting just to talk about this case. (...) and she’s there* [talking about survivor referred to the DV center]*. She is now being assisted by their team” *(Social Worker 2).

 The results from this study were used to design the intervention to be implemented in the two PHC Clinics (Table 3).

**Table 3:** Adaptation of the DV Intervention According to the Key Barriers Affecting Systems Readiness

**Building Blocks**	**Key Barriers Affecting Systems Readiness**	**Adaptations to DV Intervention Content**
Governance and leadership	DV policies are loose and broad	Provide clear information on guidelines and referral flow during training. Develop and disseminate leaflets detailing Standard Operating Procedures and care flows.
	Managers do not prioritize DV	Include/invite managers in the DV training. Discuss how to consider DV as a performance indicator in consultative committee.
	Productivity goals affect identification and consultation times for DV	Lower expectations, keep DV response simple: how to ask and how to respond and refer quickly – using role-plays.
Health workforce Service delivery Health information systems	Lack of clear professional roles	Intervention clarifies roles for all health professionals, managers and NPV teams.
	Lack of clear protocol and flows	Establish an agreed protocol and flow based on current policies and international evidence.
	Lack of empathy around women’s choices leading to blaming and pushing ‘solutions’ onto survivors using own values and beliefs	Use interactive game (‘In your shoes’ Brazilian version) and role plays to ensure healthcare providers understand women and act more person centred.
	Weakness and low visibility of NPV	Special training for the group to reinforce their role.
	Providers fear of family retaliation	Discuss infrastructure to guarantee confidentiality, safety plan for women and providers (including home visits), and clarify manager’ support in relation to safety.
	Lack of clarity regarding the mandatory report to the epidemiological surveillance	Discuss the importance of epidemiological surveillance, how to complete the form and clarify how the data is used.
Coordination	Lack of knowledge about intersectoral network and limited collaboration	Organize introductory meetings between specialized services and NPV teams.

Abbreviations: PHC, primary healthcare; NPV, violence prevention nucleus; DV, domestic violence.

## Discussion

 Our readiness assessment of 2 PHC clinics in urban Brazil has revealed gaps in both current policy and practice that need to be addressed for an effective response to DV, particularly with regards to governance and leadership, service delivery and health workforce.

 Despite the existence of a comprehensive policy framework on VAW, DV received less political recognition, being perceived as a less relevant health priority in comparison to other more traditional biomedical health issues. This may also be related with cultural aspects that contribute to the trivialisation of DV. Furthermore, the response to DV lost its visibility as it was diluted within a generic violence response.

 The lack of prioritisation of DV was also expressed in the organizational structure of the health system in São Paulo and many cities in Brazil. Based on contracts between the government and private non-profit organizations, the PHC system prioritised the numbers of medical and nurse consultations and community health agent household visits as main performance indicators, resulting in burgeoning workloads in recent years. This hindered the legitimacy of healthcare providers’ time to address DV and develop a supportive DV structure. Research about implementation of family violence policies in PHC in New Zealand also reported how the absence of a DV performance indicator was an obstacle to policy implementation.^54^ In order to challenge the invisibility of DV and legitimise the role of healthcare providers in addressing it, identification and referral of DV cases should become a key performance indicator in future PHC contracts (Table 3). However, caution is warranted as overzealous action by untrained healthcare providers may jeopardise women’s safety. Inappropriate responses to disclosure, the absence of referral pathways, and ambiguous roles may have unintended negative consequences for women experiencing abuse or even for providers themselves.

 While having a policy and a regulatory framework on VAW is crucial, political support and clarity appeared to be essential for implementation. The roles and responsibilities of central and local level managers (and providers) were not clearly defined which, in turn, affected the implementation of the policies at facility-level. The lack of clear guidance from both the central and municipal levels, including flowcharts and standard operating procedures, emerged as crucial factors that weakened DV policy implementation in the accounts of providers and managers, as also discussed elsewhere.^55^ Health managers are required to establish clear roles and care pathways (including referrals within and outside the clinic) to ensure the self-efficacy and safety of providers. Setting feasible goals, supervision and monitoring structures are also crucial for embedding new practices and ensuring that they become part of routine care. To overcome this gap, the proposed DV intervention will review - with the support of key stakeholders – the Care Pathway already in place to develop a clear flow and standard procedures (Table 3).

 These findings are not unique to Brazil.^42,56^ The presence of detailed and explicit guidance on DV has proven to be a facilitating element regarding health systems readiness in other studies.^6,8^ However, it is unclear how to build and sustain leadership skills among health managers and policy-makers, in spite of its importance. Only a few studies of other types of interventions in LMICs (mostly outside of the field of VAW) have tried to strengthen the leadership skills of health managers through mentoring and partnerships^57-59^ although with challenges. For instance, a leadership intervention for managers in Ghana failed to be institutionalised due to the lack of consideration of the organisational context and its constraints in which managers were embedded.^58^ However, providing training to both managers and health workers in primary care in South Africa led to increased recognition of intimate partner violence as a health problem and consequently, to the increased support to health workers by management.^59^ In this regard, the proposed DV intervention should also include the managers in the training process (Table 3). It must be stated, however, that public policies will depends upon the broader political landscape and the current surge of conservative ideas affect the odds of local managers prioritising a highly controversial social and health issue as DV.

 Individual-level challenges reported by providers included lack of time and knowledge of how to respond, as well as fear of dealing with DV, all of them partly related to the lack of a supportive structure, proper training and clear roles within the care pathway, in accordance with a recent systematic review^60^ on obstacles and facilitators to address DV in healthcare settings in Brazil. Despite the agency and willingness demonstrated by some providers in developing their own DV response strategies, limited management support and low priority constrained provider actions. Other Brazilian studies have shown how the implementation of DV assistance often relied upon the goodwill of some individual providers, (ie, DV champions and activists against DV) rather than on the collective effort of staff, managers and policy-makers.^37^

 In our study, providers frequently adopted a biomedical view when addressing DV, even though they recognized it was a health issue and that DV was unacceptable. This recognition did not increase provider’s self-efficacy to respond to DV in clinics – probably, due to other facility and system level constraints including unclear protocols and no in-service training that weakened the integration of a DV response.^7,46^ When acting without proper DV training and support, providers may try to “fix/cure” the issue, resulting in feelings of helplessness or frustration. This can lead to victim blaming, which may place women and themselves at risk, as reported elsewhere.^61^ We propose to use an interactive game in the training in order to enhance empathy and understanding of women constraints (Table 3).

 Adequately trained and motivated providers can facilitate integration of health interventions.^6,42,62^ DV training is thus important to help clarify providers’ values and the boundaries of their role within a comprehensive multi-sector response. The lack of knowledge and trust in the specialized DV network is another obstacle that must be tackled, considering that the knowledge of where to refer may help providers to deal better with time constraints and safety issues (Table 3).

 Readiness is a multi-faceted concept,^12^ requiring a range of interlinked system capabilities.^9^ Having DV laws and policies in place, together with willing and motivated providers can be important facilitators for systems readiness. However, these factors alone cannot overcome other system challenges that may weaken implementation of DV policies such as lack of adequate training, weak collaboration, and limited management support. Socio-cultural factors also deeply affected DV prioritisation. Although the Maria da Penha law was essential in bringing DV identification and referral to the forefront, the current government’s attack on women’s rights hindered its full implementation. Our results cut across several of the health systems building blocks, showing that it is not just one system dimension that affects readiness, but it is the interlinkage across various dimensions that allows a systems perspective.^63^ This analysis of health systems readiness informed the development of a culturally appropriate intervention, as it identified gaps in health systems to DV response, which were subsequently addressed in the intervention development phase (Table 3).

###  Limitations and Strengths

 Health system readiness assessment was important to recognise gaps in systems, service, and providers’ capacity to adopt a DV intervention and to strengthen the DV response. The readiness assessment allowed us to identify many ongoing DV initiatives and policies in place, but also to understand their gaps (and plan for such shortcomings). It was crucial to propose an intervention that recognised what had been done previously and lessons learned. With the readiness lens, we were able to design a more tailored intervention that considered the different levels of the health system, and that recognised the critical importance of working closely with managers and stakeholders.

 This study has two main limitations. The first one is the absence of women survivor’s perspective which would have helped to understand their views on acceptability and accessibility of DV care in the PHC services – which is a research focus that is often neglected when developing health interventions for providers. We interviewed women who showed great acceptability to non-judgmental approach by healthcare providers, but those results are reported elsewhere.^64^ This study also explored policy implementation and service readiness at one point in time (prior to intervention development and delivery). However, as health system readiness is an evolving process (and not static), future intervention studies could also explore key readiness dimensions during or after delivery of the intervention.’

 Although our study sample was limited to two PHC clinics in São Paulo, our findings may be applicable to other PHC settings in Brazil and other countries with similar health system and social contexts. As a research recommendation to other developing countries with similar context, we highlight the importance of better understanding the role of health managers in the implementation of complex interventions, and of considering the political and organizational context of health services.

## Conclusion

 This is the first study to assess health system readiness to implement a DV intervention in PHC in Brazil and South America. Assessing readiness is critical because it helps to assess what services and infrastructure are already in place and identify obstacles that could hinder adaptation and integration of an intervention to strengthen the response to DV before implementation. It helps reduce preparedness gaps and anticipates potential challenges to ensure effective implementation. Traditional biomedical approaches are inadequate and inappropriate for addressing the complexities of DV. Adopting a supportive, woman-centred approach is recommended and may improve the quality of PHC service provision overall.

## Ethical issues

 Ethical approval was obtained from University of São Paulo (document 2.079.832), São Paulo’s Health Department (document 2.142.949), and from collaborating UK academic institutions (London School of Hygiene & Tropical Medicine and University of Bristol). Concerns regarding the avoidance of harm to interviewees and interviewers were paramount, and the study ethical procedures were drawn upon the WHO guideline Putting Women First.^65^ Interviewers were ready to refer any healthcare provider that experienced violence, even though it was not necessary. There were also team discussions about the interviews oriented to debrief and support.

## Competing interests

 Authors declare that they have no competing interests.

## Authors’ contributions

 Conception and design: AFPLd, MC; Acquisition of data: SP, JMA, RGB, CGVG; Analysis and interpretation of data: AFPLd, LBS, MC, LJB, SP, JMA, RGB, CGVG; Drafting of the manuscript: AFPLd, SP, LBS, MC; Critical revision of the manuscript for important intellectual content: LJB, GF, LBS, MC, AFPLd; Obtaining funding: GF, LJB; Supervision: MC, GF.

## Funding

 The research was funded by the Medical Research Council Global Challenges Research Foundation Award (Grant number: MR/P02510/1). The writing up of the paper was made possible by a National Institute of Health (NIHR) (17/63/125) using UK aid from the UK Government to support global health research. The views expressed in this publication are those of the authors(s) and not necessarily those of the NIHR or the UK Government.

## Endnotes

 [1] Rice and beans are the basis of Brazilian food, this expression is equivalent to “bread and butter.”
